# Arbuscular mycorrhizal fungal communities in soils where astragalus had grown for 2 years were similar to those in the abandoned farmland

**DOI:** 10.3389/fmicb.2023.1293496

**Published:** 2024-01-04

**Authors:** Zhi-Gang An, Hu-Shan Shang, Zhi-Jia Cui, Yu-Fang Huang, Rui Wu, Run-Hong Li

**Affiliations:** ^1^College of Public Health, Gansu University of Chinese Medicine, Lanzhou, China; ^2^Pharmacy Department, Gansu University of Chinese Medicine, Dingxi, China; ^3^Chinese Herbal Medicine Institute, Dingxi Academy of Agricultural Sciences, Dingxi, China; ^4^College of Pharmacy, Gansu University of Chinese Medicine, Lanzhou, China

**Keywords:** arbuscular mycorrhizal fungi, astragalus, interannual soil, network, alpha diversity, beta diversity

## Abstract

**Purpose:**

Astragalus-cultivated soils are enriched in arbuscular mycorrhizal fungi (AMF); however, the community changes of AMF between years in stragalus-cultivated soils are still unclear.

**Methods:**

To illustrate this, using high-throughput amplicon sequencing and quantitative real-time PCR, we analyzed the AMF communities of the abandoned farmlands and interannual astragalus-cultivated soils for 1-, 2-, 3-, and 4-years, including community composition, dominant, core, specific and significantly fluctuating AMF, co-occurrence network, alpha diversity, and beta diversity.

**Results:**

A total of 74 OTUs were classified into one phylum, Glomeromycota; one class, Glomeromycetes; four orders; four families; and six genera. The 2-year soil had the highest number of reads among the interannual soils. Only one OTU was shared among all interannual soils. The treatments significantly affected the Ace, Shannoneven, and Shannon estimators of the communities. The 2-year soil had the highest richness, evenness, and diversity among all interannual soils and was the closest to the abandoned farmland in terms of alpha diversity. *Glomus* of the family Glomeraceae was the dominant genus present in all treatments, and the composition of the dominant genus in interannual soils was different. Both *Glomus* and *Diversispora* were the core AMF in interannual soils, and specific AMF existed in different interannual soils. *Glomus* is a genus that exhibits significant interannual variation. The interannual time significantly affected the network connectivity. The results of the principal coordinate analysis showed that the community composition of the interannual soils was close to each other and separated from the abandoned farmland, and that the interannual time significantly affected the community composition.

**Conclusion:**

Among the interannual soils, the 2-year soil may be more suitable for *A. sinensis* seedling rotation.

## Introduction

Astragalus (Huangqi in Chinese, also known as Astragali Radix) has a long history of medicinal and edible value in China and is the dried root of *Astragalus membranaceus* (Fisch.) Bge. var. *mongholicus* (Bge.) Hsiao and *Astragalus membranaceus* (Fiscn.) Bqe (Chen et al., 2020). Astragalus is widely utilized in Chinese medicine and the food industry because it functions to tonify Qi, strengthens the body’s immunity, and regulates blood sugar (Sun et al., 2017; Xu et al., 2022).

Dingxi in Gansu Province is an original production region for astragalus (Durazzo et al., 2021; Sheng et al., 2021). Astragalus is a perennial medicinal plant, sown in the first year and harvested in the second, third and/or fourth year (Sun et al., 2020; Huang et al., 2022). Astragalus belongs to the legume family and can form a symbiotic system with arbuscular mycorrhizal fungi (AMF), which are abundant in the soil where the host plant grows (Lin et al., 2019; Primieri et al., 2022). Dingxi is also the main cultivation area for *Angelica sinensis*. The seedlings usually cultivated in abandoned farmlands. However this approach cultivating the seedlings is not sustainable (Wang et al., 2019; Xu et al., 2020). Hence, in past studies, four types of crop-cultivated soil were tested to cultivate *A. sinensis* seedlings, and the results indicated that the *A. sinensis* seedlings cultivated in the astragalus-cultivated soil were of good quality, and we found that the astragalus-cultivated soil was a suitable soil for fostering *A. sinensis* seedlings (Jin et al., 2018; An et al., 2023). Bai et al. (2019) cultivated *A. sinensis* seedlings on a crop-cultivated field followed by pea-astragalus rotation, and the results showed that high-quality seedlings could be harvested by pea-astragalus rotation farmland. We conducted experiments on the cultivation of *A. sinensis* seedlings in both alpine meadow soils and crop-cultivated soils, with the aim of to investigating the characteristics of the fungal community composition and function within the rhizosphere of *A. sinensis* seedlings (An et al., 2020). We found that the relative abundance of AMF in astragalus-cultivated soil was higher than that in other crop-cultivated soils and alpine meadow soil, indicating that astragalus-cultivated soil was enriched with more AMF (An et al., 2023). This was one of the reasons for fostering high-quality seedlings of *A. sinensis*.

AMF are widespread root endosymbionts of terrestrial plants that play a beneficial role in sustainable agriculture. They aid in the absorption of phosphorous and nitrogen by the host, resulting in enhanced host productivity, while in turn they receive carbon sources from their hosts in exchange (Lee et al., 2013; Wang et al., 2017). The available evidence suggests that crop rotation changes the diversity and composition of AMF in the soil, and some crops increase the abundance of AMF, while others produce the opposite effect (Sosa-Hernández et al., 2019; Wang et al., 2023).

It is not clear how AMF community composition changes among the interannual soils from the cultivating seedling stage (the first year) to the forming herb stage (the second, third, and fourth years) in astragalus-cultivated soils. Therefore, we hypothesize that the AMF community composition varied significantly among the interannual soils, and that the AMF community composition of the 2-year soil was similar to that of the abandoned farmland. To test this hypothesis, we investigated the AMF community composition in interannual astragalus soils. From the perspective of AMF communities, the results will help to provide a theoretical basis for the selection of astragalus interannual soil for the cultivation of *A. sinensis* seedlings.

## Materials and methods

### Study site and experimental design

The test site was located at Dingxi Institute of Agricultural Science (N 35°58′, E 104°62′), with an altitude of 1,915 m, average annual temperature of 7.2°C, annual sunshine hours of 2,500 h, average annual frost-free period of 140 d, and average annual rainfall of over 400 mm. Field experiment design was a random complete block design. The treatments were prepared by culturing astragalus for 1, 2, 3, and 4 years, as well as the control treatment with abandoned farmland (Supplementary Figure S1). In this study, the abandoned farmland is defined as land that is not cultivated and on which a variety of plants grow freely. Each plot of astragalus cultivation was three meters by six meters. Before the test was implemented, broad bean (*Vicia faba* L.) was planted in the field and completed one growing season. We maintained consistent plot management. Each plot was cultivated by *A. membranaceus*, and the plants were harvested on schedule.

### Sample preparation

After harvesting the plants in the first, second, third, and/or fourth years, both interannual soils and abandoned 4-year farmland soils were sampled to a depth of about 20 cm using the randomized five-point sampling method. Each plot sample was mixed and packed in sterile bags, recorded, and stored. Leaves, roots, stones, and other debris were removed from the samples, and the samples were air-dried. After the four replication samples were collected, they were mixed together and transferred to sterile tubes.

### DNA extraction and amplification

DNA was extracted from the samples using E.Z.N.A.^®^ soil DNA kit (Omega). The quality of genomic DNA was examined by 1% agarose gel electrophoresis, and DNA concentration and purity were determined using a NanoDrop2000 (Thermo Scientific). Genomic DNA was used as a template for amplification using ABI GeneAmp^®^9700 PCR (ABI). Specific primer pairs (Zhu et al., 2016) with barcodes are shown in Table 1. PCR reaction system consisted of 20 μL, including 5 × FastPfu Buffer 4 μL, 2.5 mM dNTPs 2 μL, 5 μM forward primer 0.8 μL, 5 μM reverse primer 0.8 μL, FastPfu DNA polymerase (TransGen Biotech) 0.4 μL, bovine serum albumin 0.2 μL, template DNA 10 ng, and dd H_2_O to reach a final volume of 20 μL. PCR amplification conditions were as follows: initial 95°C for 3 min, denaturation 95°C for 30 s, annealing 55°C for 30 s, extension 72°C for 45 s, first amplification 32 cycles/s amplification 25 cycles, and extension 72°C for 10 min. The PCR products were detected using 2% agarose gel electrophoresis.

**Table 1:** Primer design for nested PCR.

Sequencing region	Base sequence
338F/806R	338F (ACTCCTACGGGAGGCAGCAG)
	806R (GGACTACHVGGGTWTCTAAT)
AMV4.5NF/AMDGR	AMV4.5NF (AAGCTCGTAGTTGAATTTCG)
	AMDGR (CCCAACTATCCCTATTAATCAT)

### Library construction and sequencing

PCR products from the same sample were mixed and recovered on a 2% agarose gel. The recovered products were purified using the AxyPrep DNA Gel Extraction Kit (Axygen Biosciences), detected by 2% agarose gel electrophoresis, and quantified using a Quantus^™^ Fluorometer (Promega).

The purified PCR products were used to construct the library using NEXTFLEX Rapid DNA-Seq Kit (Bioo Scientific): (i) splice linkage; (ii) removal of splice self-linked fragments using magnetic bead screening; (iii) enrichment of library template using PCR amplification; and (iv) recovery of PCR products using magnetic beads to obtain the final library. Sequencing was performed using the Illumina MiSeq PE300 platform (Shanghai Meiji Biomedical Technology Co., Ltd.). The raw data were uploaded to the NCBI SRA database (Accession: PRJNA905095).

### OTU clustering and species annotation

Raw FASTQ files were de-multiplexed using an in-house Perl script, and then quality-filtered by FASTP (Chen et al., 2018) (V 0.19.6) and merged by FLASH (Magoc and Salzberg, 2011) (V 1.2.11) as per the following criteria: (i) the 300 bp reads were truncated at any site receiving an average quality score of <20 over a 50 bp sliding window, and truncated reads shorter than 50 bp, and reads containing ambiguous characters were discarded; (ii) only overlapping sequences longer than 10 bp were assembled according to their overlapping sequence. The maximum mismatch ratio in the overlap region was 0.2. Reads that could not be assembled were discarded; (iii) samples were distinguished according to the barcode and primers, and the sequence direction was adjusted; barcodes were matched exactly, and the mismatch in primer matching was two nucleotides.

The optimized sequences were clustered into operational taxonomic units (OTUs) using UPARSE (Stackebrandt and Goebel, 1994; Edgar, 2013) (V 7.1) with a 97% sequence similarity level. Annotation for each sequence was implemented in the UNITE (V 8.0) database. The most abundant sequence for each OTU was selected as a representative sequence. The samples were normalized according to the minimum number of sample sequences. The taxonomy of each OTU representative sequence was analyzed using the RDP Classifier (Wang et al., 2007) (V 2.13) against the UNITE (Release 8.0) database with a confidence threshold of 0.7, and the community composition of each sample was counted at different taxonomic levels.

### Co-occurrence network

The OTUs correlation matrix of interannual soils was studied by selecting the top 50 species in terms of total abundance at the genus level and calculating Spearman’s correlation coefficients (−0.5 ≤ *r* ≤ 0.5, *p* < 0.05) between species. NetworkX was used to construct and analyze the co-occurrence network.

### Quantitative real-time PCR and absolute abundance

Primer pairs AMV4.5NF/AMDGR were used to determine AMF abundance by ABI 7300 ABI7300 Fluorescent Quantitative PCR System (Applied Biosystems, United States). The standard curve was constructed with the plasmid vector (pMD18-T, 2692 bp). Each PCR reaction was carried out in a 20 μL qPCR reaction mixture containing 10 μL ChamQ SYBR Color qPCR Mix (2×) (Vazyme Biotech, China), 0.8 μL PCR forward and reverse primers (both 5 μM), 0.4 μL X ROX Reference Dye1, 2 μL DNA template, and 6 μL double distilled water (dd H_2_O). Quantitative real-time PCR reactions were set to 95°C for 3 min, followed by 40 cycles of 95°C for 5 s, 58°C for 30 s, and 72°C for 1 min. Analysis of amplification and melting curves for AMF gene quantification showed excellent specificity of the qPCR with an efficiency of 96.44%. AMF gene standard curve $y=-3.4104\;x+40.509\;\left(R^{2}=0.9988\right)$ .

To allow for a quantitative comparison, the relative abundance values of specific taxa were converted into absolute abundance values by multiplying by the corresponding quantitative fluorescence values (copies × 10^6^/g).

### Statistical analyses

ACE and Shannon indices were calculated using MOTHUR (Schloss et al., 2009) (http://www.mothur.org/wiki/Calculators). Data were analyzed with principal coordinate analysis (PCoA) based on Bray–Curtis, Anosim test (permutations 999), Kruskal–Wallis rank sum test (multiple test corrected fdr and *post-hoc* test Scheffe), and One-ANOVA analysis with Tukey multiple comparisons. Statistical analyses were performed using the Meguiar’s BioCloud platform (https://cloud.majorbio.com) and Origin (2022).

## Results

### AMF community

The dilution curve results showed that the number of OTUs in all samples flattened out with increasing sequencing numbers; therefore, the amount of sequencing data was large enough to reflect the AMF diversity information in the samples (Supplementary Figure S2). Pan-OTU is the total number of species found in all samples, and core-OTU is the number of species shared by all samples. As the sample size increased, the pan-OTUs showed an increasing upward trend (Supplementary Figure S3A), whereas the core-OTUs showed a decreasing trend (Supplementary Figure S3B).

**Figure 1:**
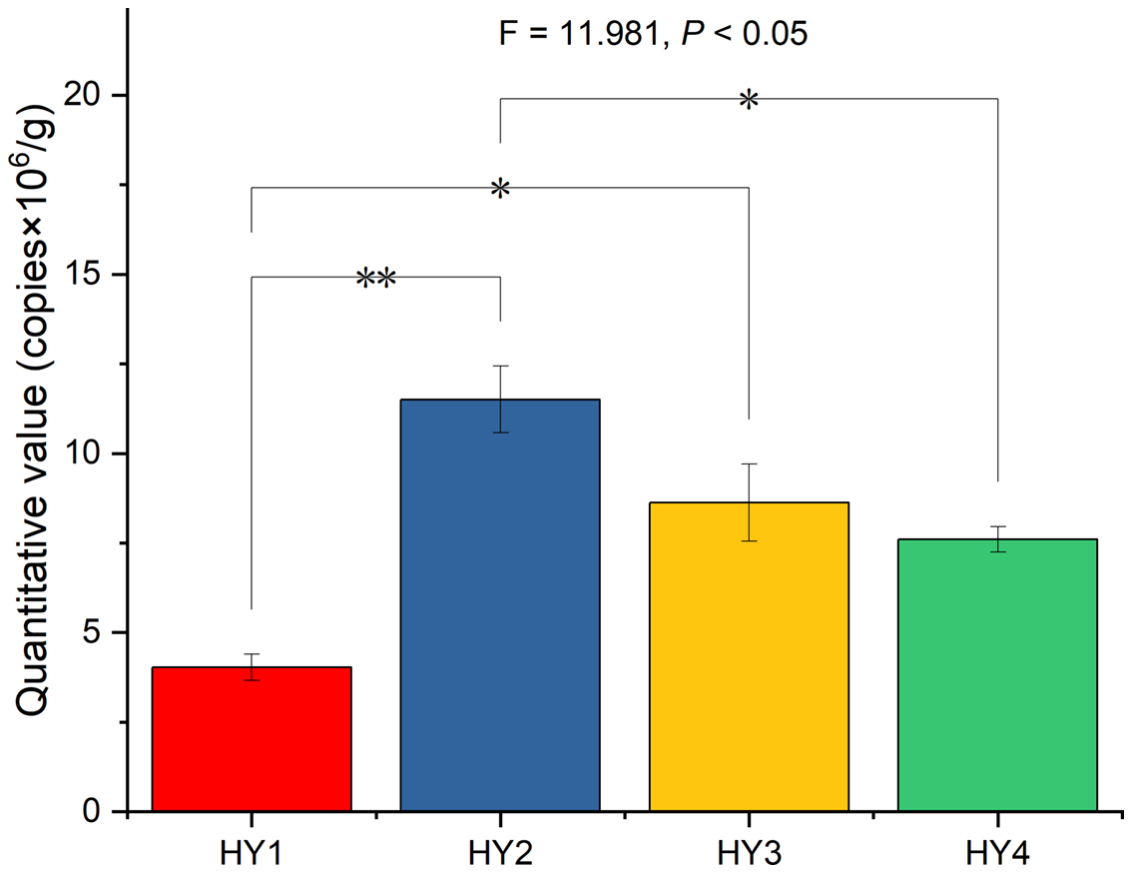
Effect of interannual soil treatment on absolute quantitative values based on One-ANOVA with Tukey tests, * *p* ≤ 0.05 and ** *p* ≤ 0.01. HY1, 1-year soil; HY2, 2-year soil; HY3, 3-year soil; HY4, 4-year soil.

After splicing, filtering, and removing chimeras from the raw reads of 20 samples, 513,233 reads were obtained with 89,479 reads for 1-year soil, 115,574 reads for 2-year soil, 99,396 reads for 3-year soil, and 99,293 reads for 4-year soil. The 2-year soil had the highest number of reads. The fluorescence quantification results showed that the interannual soil treatment significantly affected the absolute abundance of AMF, with 2-year soil being significantly higher than the 1- and 4-year soils (Figure 1).

To minimize the effects of sequencing depth on alpha and beta diversity measures, the number of sequences from each sample was rarefied to 19,814 according to the minimum number of sample sequences. A total of 74 OTUs were obtained by species annotation and were classified into the phylum Glomeromycota, four orders, four families, six genera, and 27 species (Table 2). At the order level, the treatments affected the distribution of species, with only the order Glomerales present in all treatments and the variability in the distribution of the other three orders (Table 3).

**Table 2:** Taxonomic of the species annotation to OTUs.

Order	Family	Genus	Species
Diversisporales Glomerales Paraglomerales unclassified_c_Glomeromycetes	Diversisporaceae Diversisporaceae Paraglomeraceae unclassified_c_Glomeromycetes	*Diversispora* *Glomus*_f_Diversisporaceae *Glomus*_f_Glomeraceae *Paraglomus* unclassified_c_Glomeromycetes unclassified_f_Diversisporaceae	*Glomus* acnaGlo2 VTX00155 *Glomus* Glo32 VTX00124 *Glomus* Glo3 VTX00074 *Glomus* group B *Glomus* Glo G8 VTX00340 *Glomus* group B *Glomus* lamellosu VTX00193 *Glomus intraradices* VTX00105 *Glomus* MO G18 VTX00064 *Glomus* MO G20 VTX00143 *Glomus* MO G22 VTX00125 *Glomus* MO G23 VTX00222 *Glomus* MO G4 VTX00166 *Glomus* MO G7 VTX00199 *Glomus mosseae* VTX00067 *Glomus* sp. VTX00234 *Glomus* sp. VTX00301 *Glomus* sp. VTX00304 *Glomus* VeGlo18 VTX00342 *Glomus versiforme* VTX00061 *Glomus viscosum* VTX00063 *Glomus* Whitfield type 17 VTX00195 *Glomus* Wirsel OTU16 VTX00156 *Paraglomus* Glom 1B.13 VTX00308 unclassified_c_Glomeromycetes unclassified_f_Diversisporaceae unclassified_g_*Diversispora* unclassified_g_*Glomus*_f_Glomeraceae unclassified_g_*Paraglomus*

**Table 3:** Distribution of AMF in the interannual soils at Order level.

Order	CK	HY1	HY2	HY3	HY4
Diversisporales	×	✓	✓	✓	✓
Glomerales	✓	✓	✓	✓	✓
Paraglomerales	×	×	×	✓	×
unclassified_c_Glomeromycetes	×	✓	✓	×	×

✓ means present, and × means not present.

### Community composition and alpha diversity

**Figure 2:**
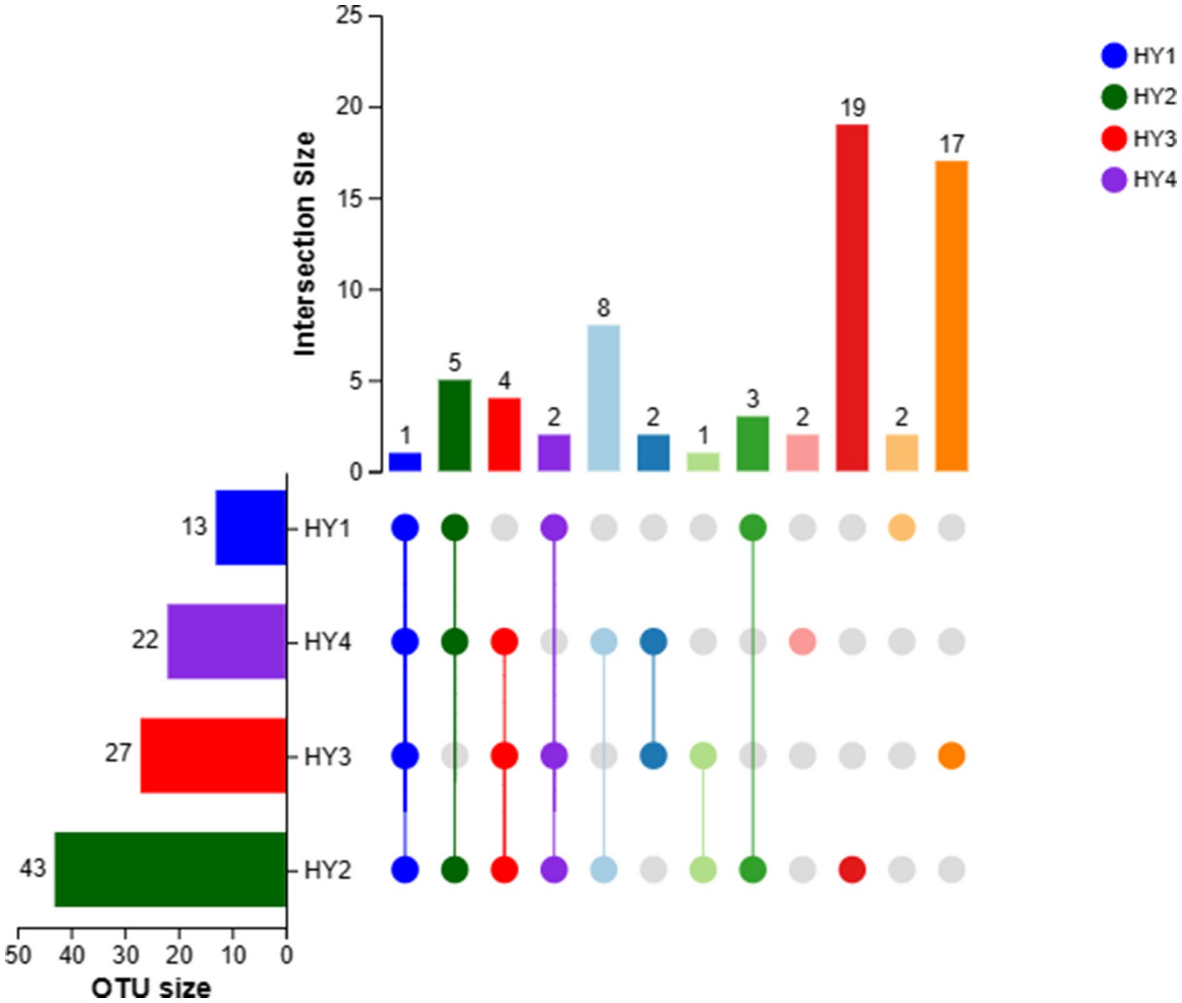
UpSet of OTUs in interannual soils. The meanings of HY1, HY2, HY3, and HY4 were illustrated in Figure 1.

Only one OTU was shared among the interannual soil samples (Figure 2). The 2-year soil had the highest number of OTUs (43 OTUs), followed by the 3-, 4-, and 1-year soils. The 2-year soil had the highest number of unique OTUs (19 OTUs), the 3-year soil had unique 17 OTUs, and the 1- and 4-year soils had two unique OTUs. Thus, the 2-year soil exhibited a rich diversity of AMF species.

**Figure 3:**
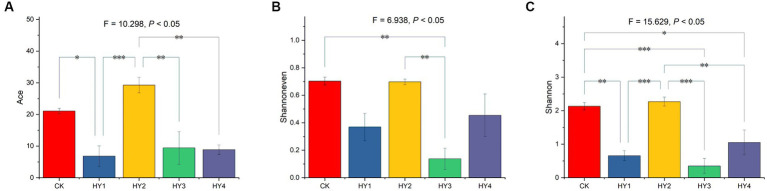
Comparisons of Ace **(A)**, Shannoneven **(B)**, and Shannon **(C)** were based on One-ANOVA with Tukey tests, * *p* ≤ 0.05, ** *p* ≤ 0.01, and *** *p* ≤ 0.001. The content of each estimator is expressed as mean ± SE (*n* = 4). CK, abandoned farmland; the meanings of HY1, HY2, HY3 and HY4 were illustrated in Figure 1.

Alpha diversity was used to describe the abundance and diversity of a community, including the Ace estimator for community richness, Shannoneven estimator for community evenness, and Shannon estimator for community diversity. One-ANOVA analysis with Tukey tests was used to investigate the Ace, Shannoneven, and Shannon of the communities. The treatments significantly affected the Ace estimator (Figure 3A). The Ace of 1-year soil was significantly lower than that of the abandoned farmland, and the Ace of 2-year soil was of no difference to that of the abandoned farmland. The Ace of the 1-, 3-, and 4-year soils were significantly lower than that of the 2-year soil, which was with the highest Ace. The treatments significantly affected the Shannoneven estimator (Figure 3B). The Shannoneven of the 3-year soil was significantly lower than that of 2-year soil and that of the abandoned farmland. There was no significant difference for Shannoneven between abandoned farmland and 2-year soil. The Shannoneven of the 3-year soil was significantly lower than that of the 2-year soil. The 2-year soil was with the highest Shannoneven in the interannual soils. Also, the treatments significantly affected Shannon estimator (Figure 3C). The Shannon of the 1-, 3-, and 4-year soils were significantly lower than that of the abandoned farmland, and the Shannon of 2-year soil was of no difference to that of the abandoned farmland. The Shannon of the 1-, 3-, and 4-year soils were significantly lower than that of the 2-year soil, which was with the highest Shannon. In conclusion, the 2-year soil had the highest Ace richness, Shannoneven evenness, and Shannon diversity in the interannual soils, and was the closest to the abandoned farmland in terms of alpha diversity.

### Dominant, core, specific, and significantly fluctuating AMF

**Figure 4:**
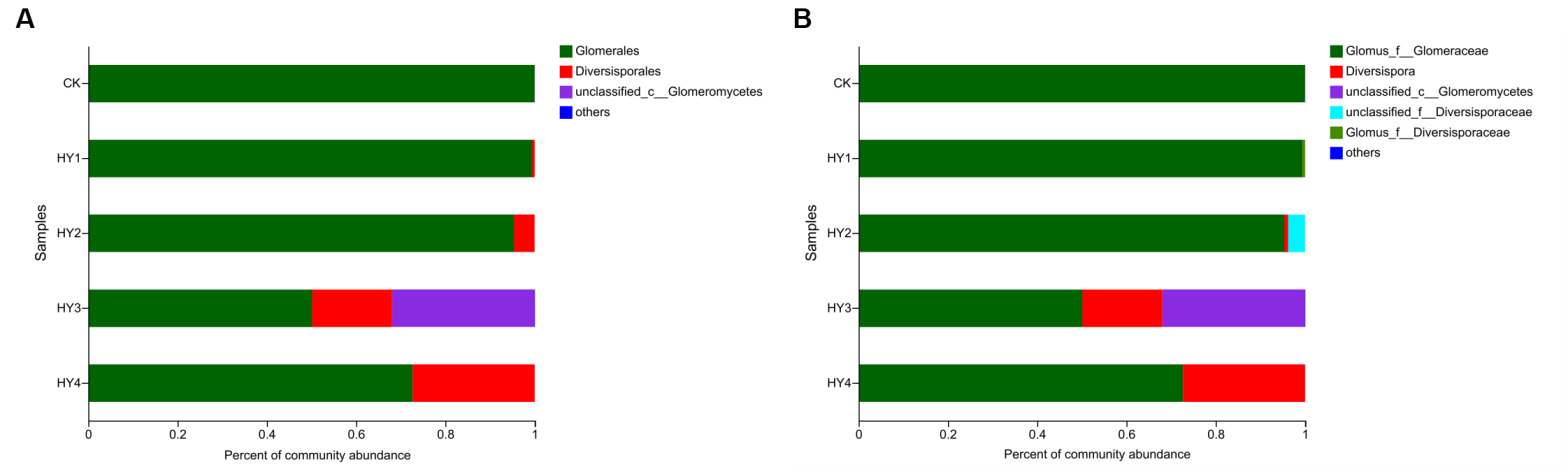
Community barplots of the relative abundances at order level **(A)** and genus level **(B)**. The meanings of CK, HY1, HY2, HY3, and HY4 were illustrated in Figure 3. f stands for family, and c for class.

The order Diversisporales was more frequently present to all interannual soils compared to the abandoned farmland (Figure 4A). At the genus level (Figure 4B), *Diversispora* was present in all interannual soils, and its abundance increased with interannual time. In this study, the genus with relative abundance (RA) of ≥1% was considered the dominant genus. The composition of the dominant genera differed among interannual soils (Table 4). The genus *Glomus* of the family Glomeraceae was present in all treatments with a relative abundance of more than 50%, while its relative abundance was more than 90% in the one- and 2-year soils. *Diversispora* was the dominant genus in the 3- and 4-year soils.

**Table 4:** Compositions of the dominant genus among interannual soils.

Treatments	Dominant genus
CK	*Glomus* of family Glomeraceae (99.97%)
HY1	*Glomus* of family Glomeraceae (99.36%)
HY2	*Glomus* of family Glomeraceae (95.31%), unclassified of family Diversisporaceae (3.80%)
HY3	*Glomus* of family Glomeraceae (50.09%), unclassified of class Glomeromycetes (31.95%), *Diversispora* (17.95%)
HY4	*Glomus* of family Glomeraceae (72.67%), *Diversispora* (27.32%)

**Figure 5:**
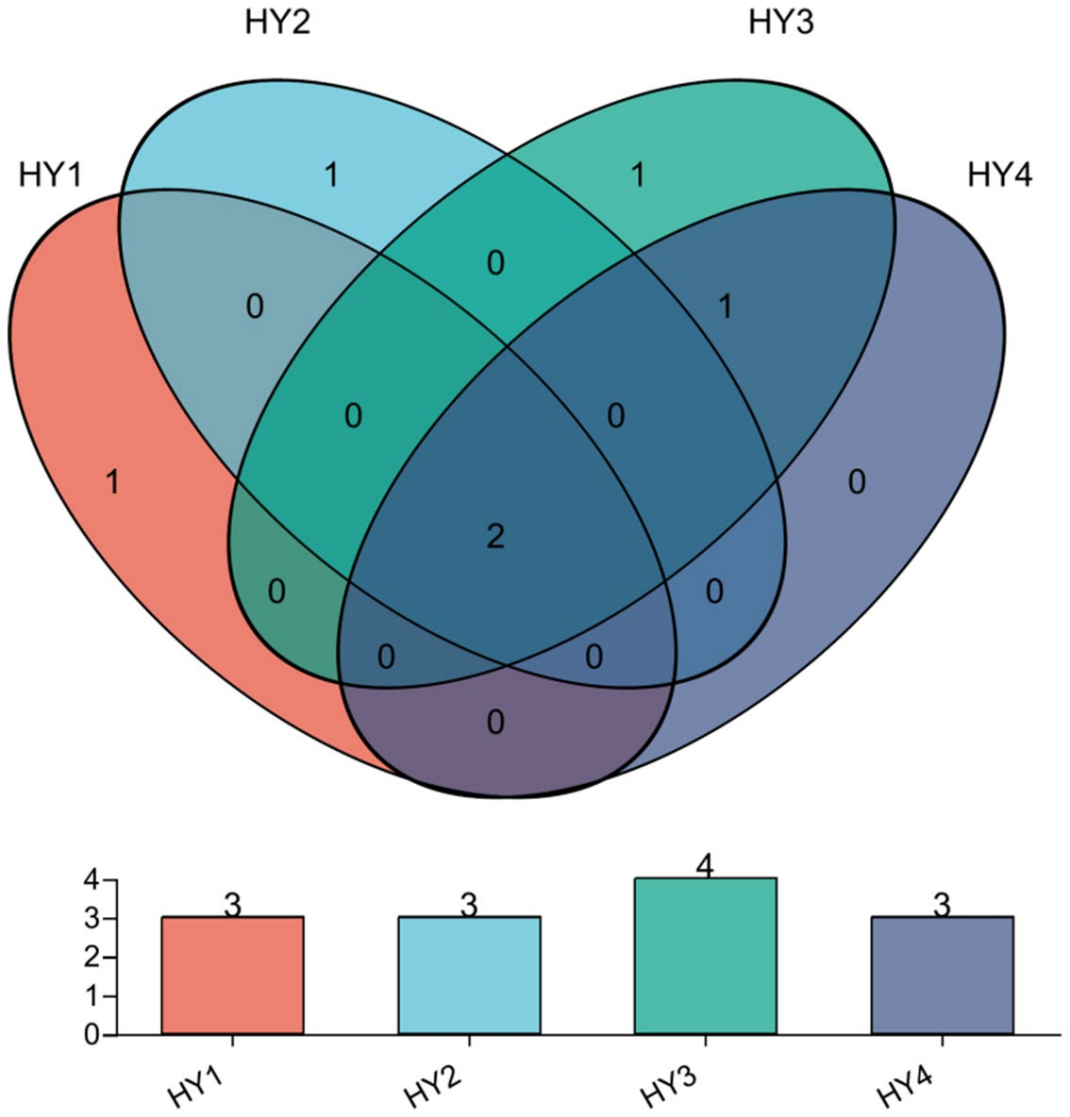
Venn diagrams at Genus level on the interannual soils. The meanings of HY1, HY2, HY3, and HY4 were illustrated in Figure 1.

The core AMF in interannual soils were identified by Venn analysis (Figure 5), and they were the genera *Glomus* and *Diversispora*. In this study, specific AMF are those that are unique in one treatment and do not appear in others. There was one specific genus *Glomus* (RA < 1%) in the 1-year soil. The specific genus for the 2-year soil was an unclassified genus of the family Diversisporaceae, and it was the dominant genus in the 2-year soil. The specific genus for the 3-year soil was *Paraglomus* (RA < 0.1%), whereas no specific genus was present in the 4-year soil.

**Figure 6:**
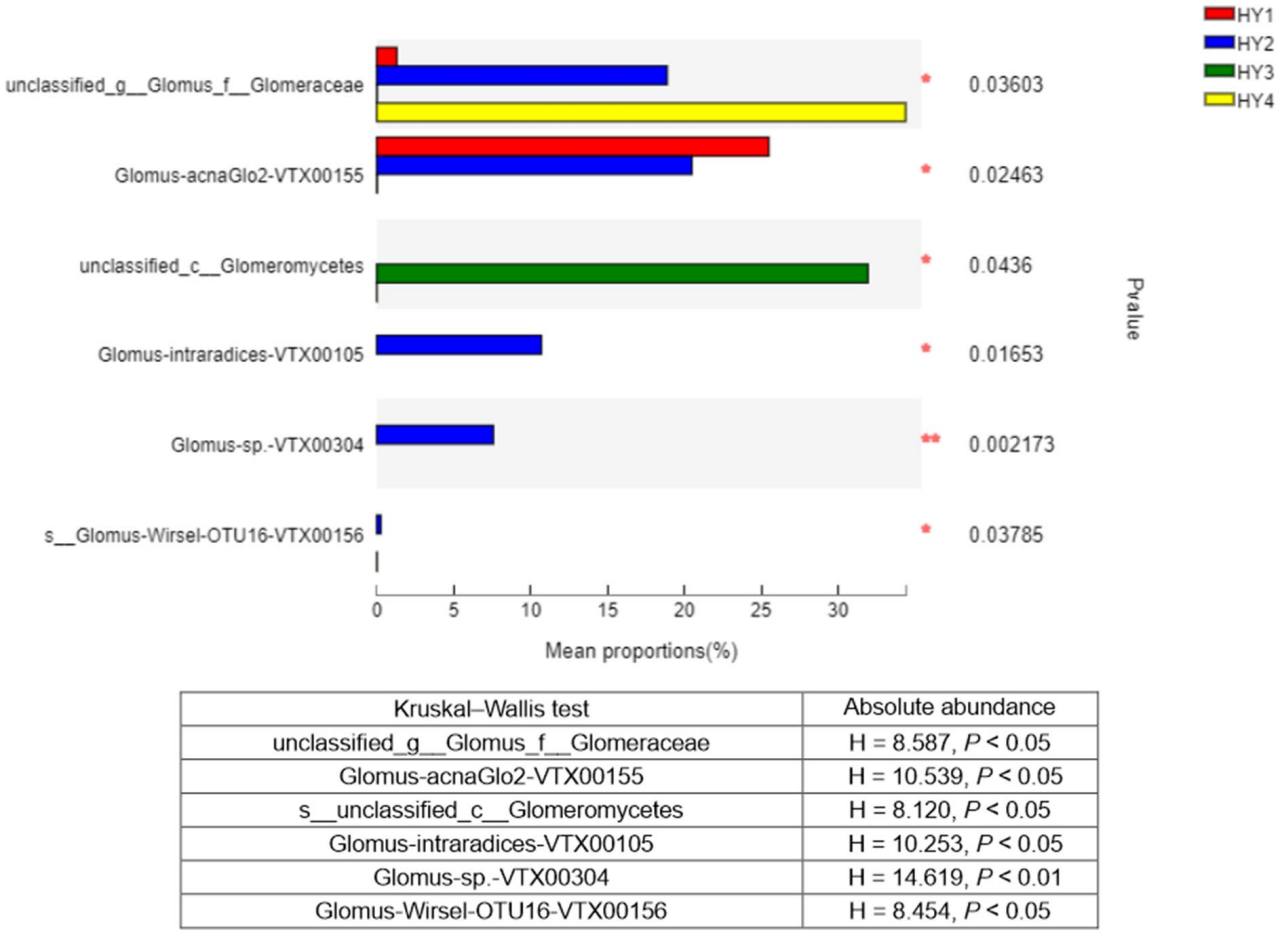
Kruskal-Wallis H test bar plot of species that differed significantly among interannual soils. Y-axis indicated species name at species level, X-axis indicated the mean relative abundance of species, and colored bars indicated different groups. *p ≤ 0.05 and **p ≤ 0.01. The meanings of HY1, HY2, HY3, and HY4 were illustrated in Figure 1. Kruskal-Wallis statistics based on the absolute abundance were shown in the table. g stands for genus, f for family, and c for class.

The Kruskal–Wallis test was used to analyze the species that showed significant differences among the interannual soils. Six species showed significant differences in relative abundance among the interannual times, and these results were consistent with those of absolute abundance (Figure 6). Thus, genus *Glomus* varied significantly among the interannual soils. Except for the species (unclassified_class_Glomeromycetes), species with significant variations in relative abundance were mainly found in the order Glomerales.

### Co-occurrence network and beta diversity

The community co-occurrence networks of interannual soils reflected relationships between species under specific environmental conditions (Supplementary Figure S4). One-ANOVA analysis of the network degree showed that the interannual time significantly (*p* < 0.01) affected the connectivity of networks (HY3^a^ > HY2^b^ > HY4^bc^ > HY1^c^; lowercase letters in the right superscript indicate the results of Tukey multiple comparisons, *p* < 0.05). The network connectivity of the 3-year soil was significantly higher than that of other soils; and its clustering coefficient was the largest, indicating that the interrelationship among species in the 3-year soil was the strongest.

**Figure 7:**
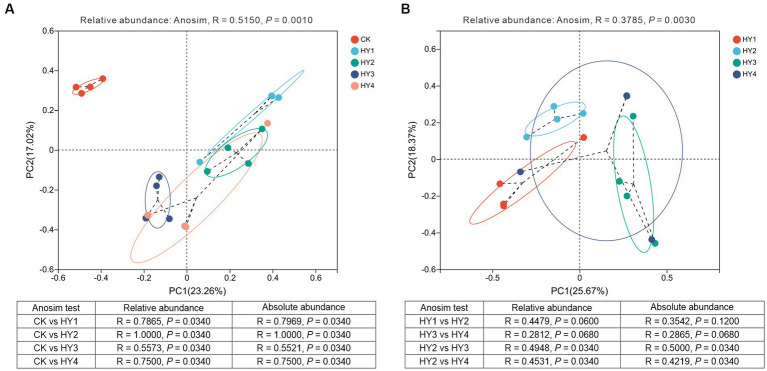
Principal coordinate analyses of the community compositions to treatments **(A)** and interannual soils **(B)** based on OUT level. Anosim statistics based on the relative and absolute abundance were shown in the table. R, degree of explanation of the difference between samples; *P*, significant test value. The meanings of CK, HY1, HY2, HY3, and HY4 were illustrated in Figure 3.

Beta diversity was used to analyze the diversity of the community along the environmental gradient. PCoA analysis was used to investigate the effect of treatments and interannual times on the community composition. The results showed that the community compositions of the interannual soils were close to each other and separated from the abandoned farmland, indicating that the community compositions of the interannual soils were different from those of the abandoned farmland (Figure 7A). Anosim test based on relative adundance (*R* = 0.5150, *p* = 0.0010) showed that the treatments significantly affected the community composition, and the consistent result was supported by Anosim test based on absolute abundance (*R* = 0.4938, *p* = 0.0010). The community compositions of the 1-year soil, 2-year soil, 3-year soil, and 4-year soil were significantly different from those of the abandoned farmland, and these results were supported by the statistical validation based on the relative and absolute abundance (Figure 7A).

The interannual times significantly affected the community composition, which was supported by Anosim test based on the relative abundance (*R* = 0.3785, *p* = 0.0030) and absolute abundance (*R* = 0.3550, *p* = 0.0030) (Figure 7B). The Anosim test showed no significant difference between the community composition of the 1-year soil and the 2-year soil, and between the 3-year soil and the 4-year soil, while the community composition of the 2-year soil was significantly different from that of the 3-year soil and the 4-year soil. These results based on the relative and absolute abundance were consistent (Figure 7B). Thus, significant changes in the community composition among interannual soils occurred in the 3-year soil.

## Discussion

In this study, we investigated the effect of interannual time on the AMF community in astragalus soils using the Illumina sequencing technology and quantitative real-time PCR. The amount of sequencing data reflected the AMF diversity in the samples. A total of 74 OTUs were identified by species annotation. The 2-year soil had the highest number of reads among the interannual soils, which was consistent with the results of AMF absolute abundance based on fluorescence quantification. This evidence strongly suggests that 2-year soil was highly enriched with AMF. All AMF belonged to the class Glomeromycetes in the phylum Glomeromycota; however, the distribution of AMF at the order level showed variability, with the order Glomerales occurring in all interannual soils.

The distribution characteristics of OTUs indicated that the 2-year soil had rich species diversity, and the Shannon analysis concluded that the diversity of the 2-year soil was significantly higher than that of the 1-, 3-, and 4-year soils. The results of both the analyses were consistent. In a 3-year continuous crop study, AMF diversity was found to be significantly higher in a 3-year continuous soil than in a 2-year soil, concluding that the continuous crop was beneficial to the accumulation of AMF diversity (Cui et al., 2018). This study showed that the interannual soils significantly affected the ACE of the community; similar results were found in a study of soybean continuous crop years, where the relative abundance of AMF communities varied significantly among soybean continuous crop soils (Cui et al., 2018). However, in all interannual soils, the maximum richness, evenness, and diversity of the community occurred in the 2-year soil, indicating that the 2-year continuous astragalus crop contributed to the increase in richness, evenness, and diversity of the AMF community.

Ace, Shannoneven, and Shannon estimators decreased significantly from 2-year soil to 3-year soil; however, the results of the beta diversity showed that the 3-year soil was the year in which significant changes in community composition occurred, thus indicating that the AMF community composition changed significantly during the third year of continuous astragalus cropping. In conclusion, the community composition of the 2-year soil had highest richness, evenness, and diversity among interannual soils, and all of them were close to the richness, evenness, and diversity of the abandoned farmland. In a previous study, we found that *A. sinensis* seedlings grown in Astragalus-cultivated soils were of good quality and the AMF abundance was higher than other soils (An et al., 2023). Therefore, from the perspective of AMF community, soil with 2 years of continuous astragalus may be more suitable for *A. sinensis* seedlings rotation. Many studies have shown that implementing a rational crop rotation strategy can increase the abundance, diversity, and composition of AMF in the soil. For example, the abundance of AMF was increased through wheat (Higo et al., 2013) and maize (Moitinho et al., 2020) rotation.

The genus *Glomus* had a high relative abundance in all treatments and was the dominant genus, whereas the genus *Diversispora* dominated in the 3- and 4-year soils. The genus *Glomus* has been found to be dominant in AMF communities within soils and rhizosphere in many studies (Xiao et al., 2022), including those conducted on soybean continuous cropping soils (Cui et al., 2018), rhizosphere of *Atractylodes lancea* in the Chongqing region (Cao et al., 2020), and rhizosphere of *Pinellia ternate* in Hangzhou and Guiyang (Shi et al., 2017). The dominant AMF genera in the potato rhizosphere in central Inner Mongolia included *Glomus* and *Diversispora* depending on the sampling site (Zhang et al., 2020). However, Lin et al. (2019) studied AMF diversity in the soils of karst habitats, and the results showed that *Rhizophagus* was the dominant genus. Chen et al., 2022 studied AMF diversity in cotton growing regions in Xinjiang, and successfully iedentied AMF spores based on morphological and molecular examinations. They found that *Paraglomus* was the dominant genus. In addition, the genera *Glomus* and *Diversispora* were the core AMF in all interannual soils. 1-, 2-, and 3-year soils possessed their own unique genera, and this may be due to the distinct ecological niches caused by specific habitats, or may be attributed to species drift (Dini-Andreote and Raaijmakers, 2018). *Glomus intraradices* has been extensively studied, and now this species belongs to the genus *Rhizophagus*, known as *Rhizophagus intraradices* (Reference URL: https://invam.ku.edu/). Inoculation with *G*. *intraradices* improved phosphorus uptake in the phosphorus-deprived state of olive trees (Jiménez-Moreno et al., 2018), and another study showed that inoculation with *G*. *intraradices* increased root colonization of *Panax ginseng*, increased the content of monomeric and total ginsenosides, and improved root activity as well as polyphenol oxidase and peroxidase activities (Tian et al., 2019). In this study, the higher abundance of *G*. *intraradices* in the 2-year soil may have favored the growth of rotational crops.

Alpha and beta diversities were used to analyze the characteristics of AMF communities on a macro level, and co-occurrence networks can reflect the relationship between community individuals at the micro level. Although the 3-year soil was the year in which the richness, evenness, and diversity of the AMF community decreased and the composition changed significantly, the network analysis of the 3-year soil showed that the relationship between the community individuals became closer. This suggests that there is an inevitable relationship between changes in species interrelationships and changes in community alpha and beta diversities.

## Conclusion

This study reported the variation in AMF communities among interannual astragalus soils. The *Glomus* was the dominant genus present in all treatments, and the composition of the dominant genus in interannual soils was different. AMF community composition varied significantly among the interannual soils. The community composition of the 2-year soil was significantly different from that of the three and 4-year soils. Significant changes in the community composition among interannual soils occurred in the 3-year soil. Although a significant difference in beta diversity between the 2-year soil and abandoned farmland was noted, the Ace, Shannoneven, and Shannon estimators in the 2-year soil were the closest to the abandoned farmland. Therefore, among the interannual soils, the 2-year soil may be more suitable for *A. sinensis* seedlings rotation.

After thoroughly analyzing the results, we identified three questions that warrant further investigation. For example, (1) why did the AMF community within the 2-year soil exhibit the highest richness among all samples? (2) why was the abundance of wasteland soils higher than expected? (3) why did a significant shift in the microbial community composition occur within the 3-year soil?

## Data availability statement

The datasets presented in this study can be found in online repositories. The names of the repository/repositories and accession number(s) can be found at: NCBI - PRJNA905095 (https://www.ncbi.nlm.nih.gov/search/all/?term=PRJNA905095).

## Author contributions

Z-GA: Formal analysis, Software, Writing – original draft, Writing – review & editing. H-SS: Data curation, Funding acquisition, Writing – review & editing. Z-JC: Data curation, Methodology, Writing – original draft. Y-FH: Methodology, Writing – original draft. RW: Investigation, Writing – original draft. R-HL: Resources, Writing – original draft.
