# Supplementary material for: Arbuscular mycorrhizal fungal communities in soils where astragalus had grown for 2 years were similar to those in the abandoned farmland
Source: Front Microbiol. 2024 Jan 4;14:1293496. doi: 10.3389/fmicb.2023.1293496 (PMC10794390; doi:10.3389/fmicb.2023.1293496)
Supplement: Supplementary file 1 [file Data_Sheet_1.pdf]

Supplementary Figure S1 A field experiment was designed with four blocks and an abandoned farmland. Each block included four treatments.

Supplementary Figure S2 Rarefaction curves of samples.

Supplementary Figure S3 Pan-OTUs(a) and core-OTUs(b). CK, abandoned farmland; HY1, 1-year soil; HY2, 2-year soil; HY3, 3-year soil; HY4, 4-year soil.

Supplementary Figure S4 Co-occurrence networks of the communities on HY1(a), HY2(b), HY3(c), and HY4(d). The filled colors in the nodes indicated the Genus level. The size of the node indicated the relative abundance of species. The red line was a positive correlation and the green line is a negative correlation. The thickness of the line indicated the correlation coefficient. Nodes, the number of nodes in a network. Degree, the network connectivity. Clustering, the network clustering coefficient. The values of Degree and Clustering were represented as the mean. HY1, 1-year soil; HY2, 2-year soil; HY3, 3-year soil; HY4, 4-year soil.
