# Supplementary figures and images for: Arbuscular mycorrhizal fungal communities in soils where astragalus had grown for 2 years were similar to those in the abandoned farmland
Source: Front Microbiol. 2024 Jan 4;14:1293496. doi: 10.3389/fmicb.2023.1293496 (PMC10794390; doi:10.3389/fmicb.2023.1293496)

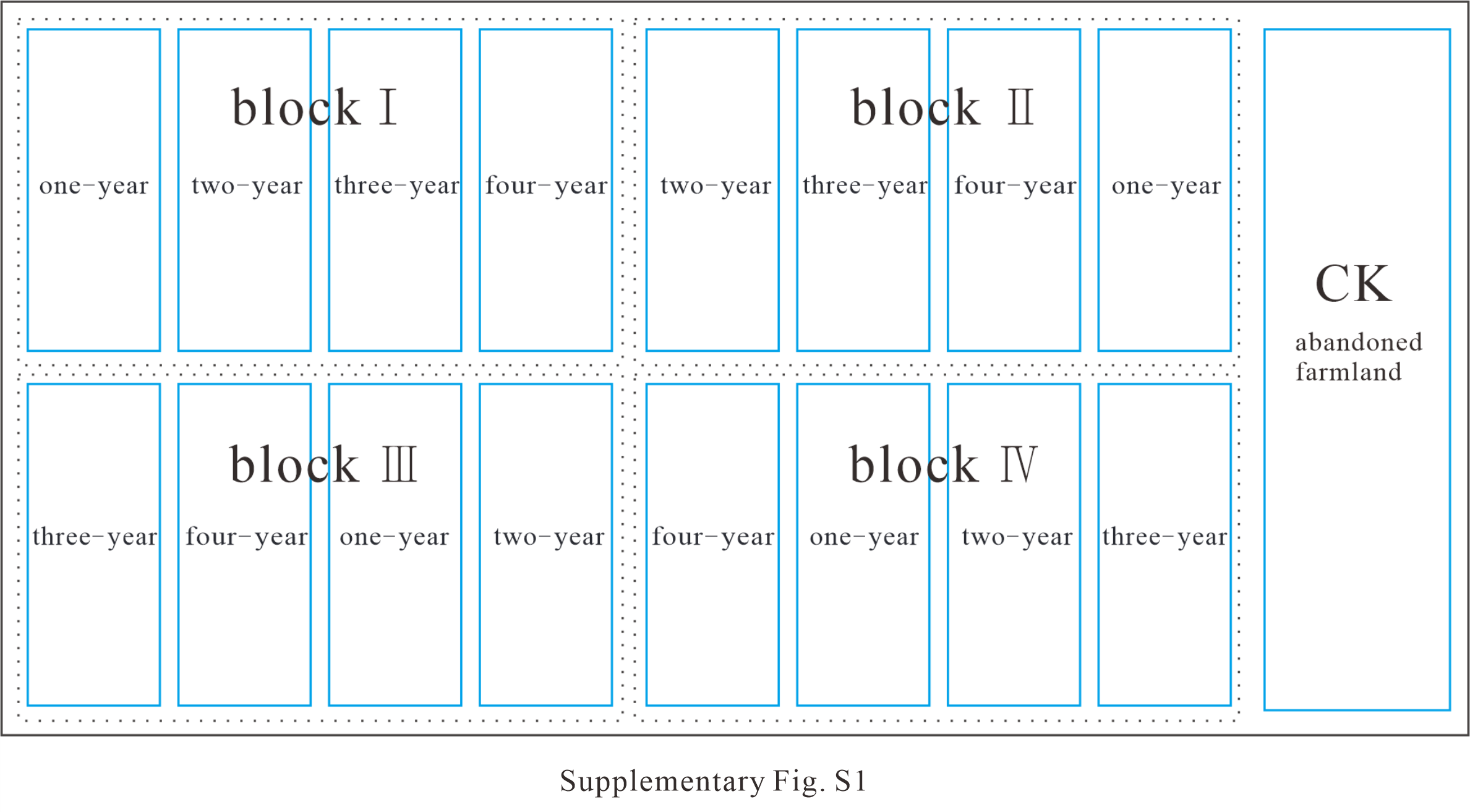

Supplement: Supplementary file 2 [file Image_1.png]

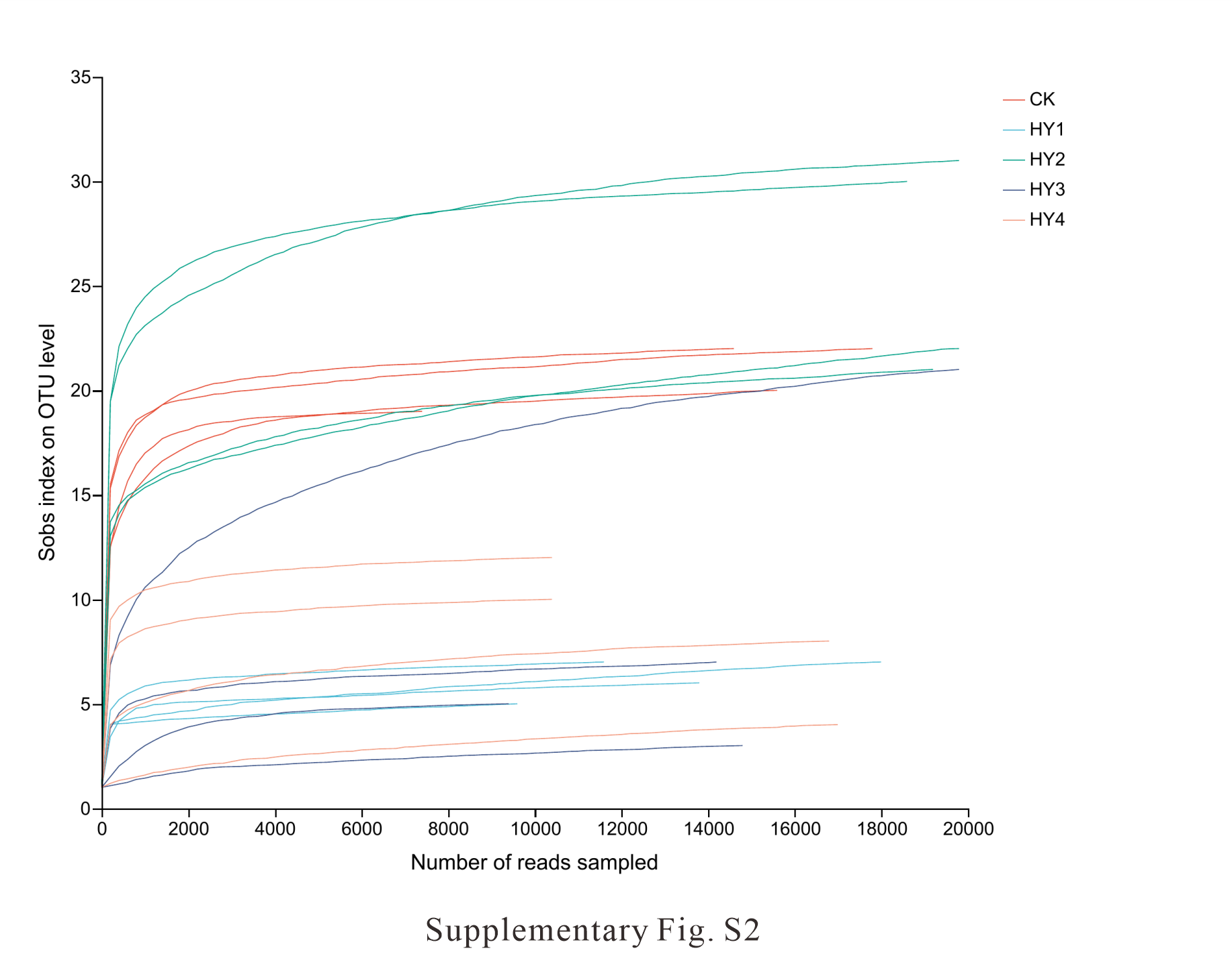

Supplement: Supplementary file 3 [file Image_2.png]

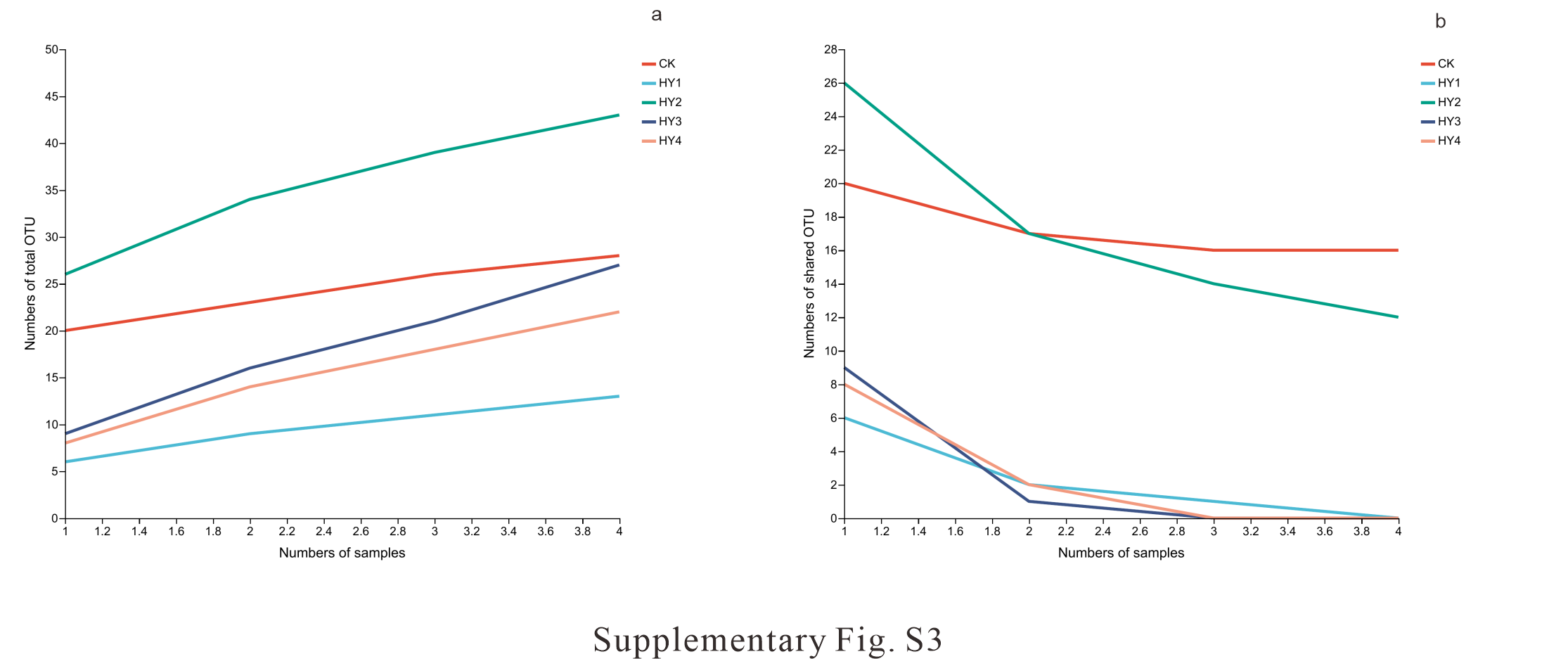

Supplement: Supplementary file 4 [file Image_3.png]

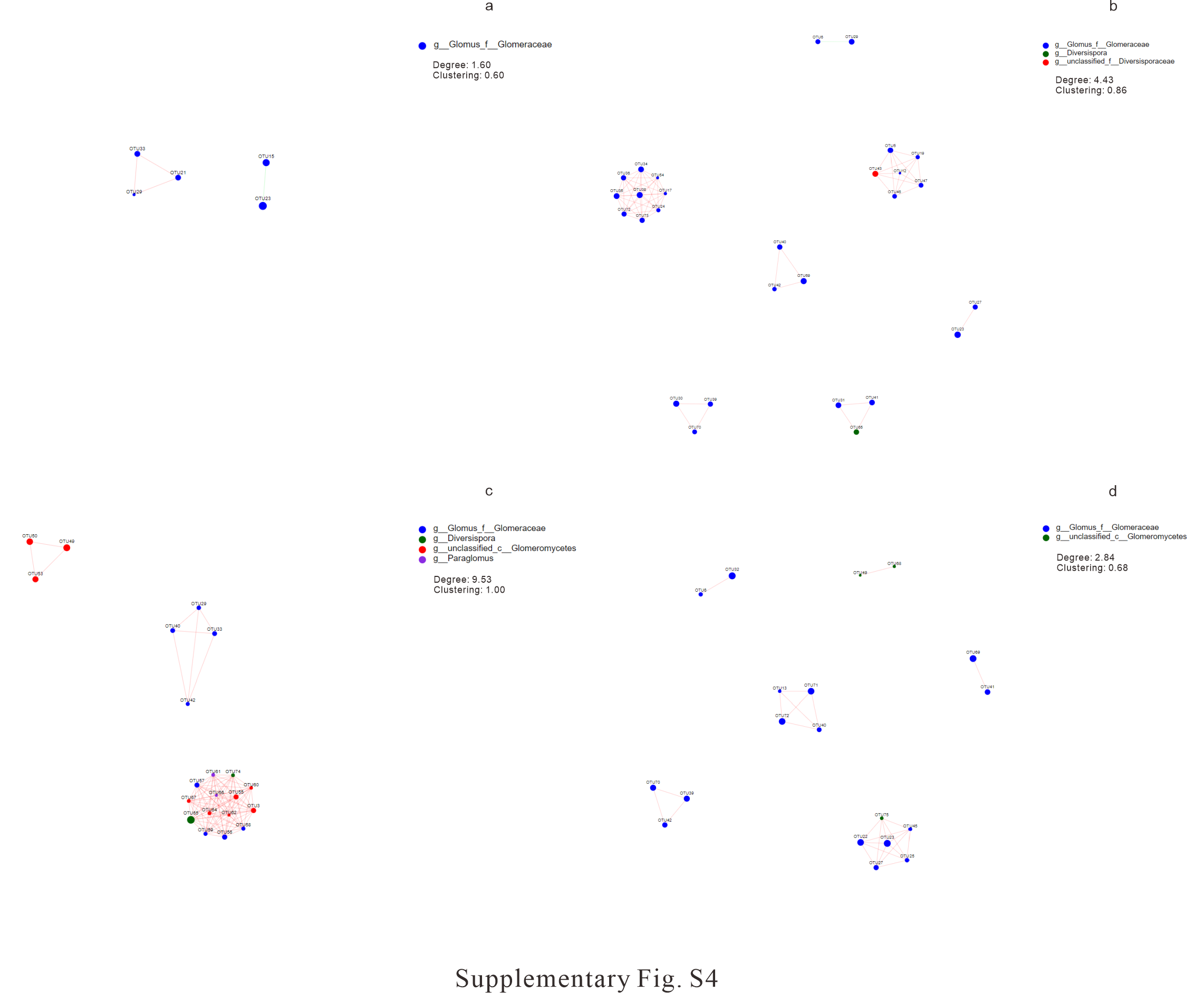

Supplement: Supplementary file 5 [file Image_4.png]
